# Non-Covalent Organocatalyzed Domino Reactions Involving Oxindoles: Recent Advances

**DOI:** 10.3390/molecules22101636

**Published:** 2017-09-29

**Authors:** Tecla Gasperi, Martina Miceli, Jean-Marc Campagne, Renata Marcia de Figueiredo

**Affiliations:** 1 Dipartimento di Scienze, Sezione di Nanoscienze e Nanotecnologie, Università degli Studi Roma Tre, via della Vasca Navale 79, I-00146 Roma, Italy; martina.miceli@icloud.com; 2 ICGM-Institut Charles Gerhardt Montpellier, UMR 5253 CNRS-UM-ENSCM, Ecole Nationale Supérieure de Chimie, 8, Rue de l’Ecole Normale, 34296 Montpellier CEDEX 5, France; jean-marc.campagne@enscm.fr

**Keywords:** spirooxindole derivatives, non-covalent organocatalysis, hydrogen-bonding, cascade, tandem, domino, enantioselectivity, chiral building blocks

## Abstract

The ubiquitous presence of spirooxindole architectures with several functionalities and stereogenic centers in bioactive molecules has been appealing for the development of novel methodologies seeking their preparation in high yields and selectivities. Expansion and refinement in the field of asymmetric organocatalysis have made possible the development of straightforward strategies that address these two requisites. In this review, we illustrate the current state-of-the-art in the field of spirooxindole synthesis through the use of non-covalent organocatalysis. We aim to provide a concise overview of very recent methods that allow to the isolation of unique, densely and diversified spirocyclic oxindole derivatives with high structural diversity via the use of cascade, tandem and domino processes.

## 1. Introduction

**Figure 1:**
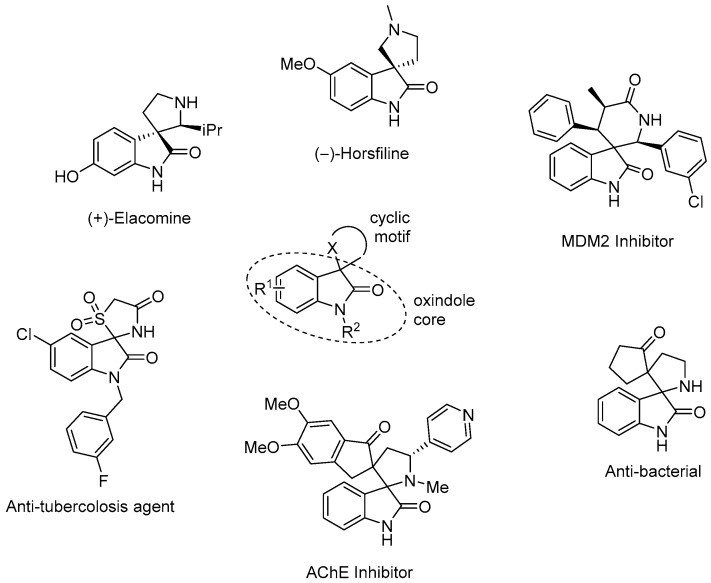
Examples of bioactive compounds built around the oxindole framework.

Heterocyclic compounds are found in a broad range of bioactive molecules, natural products, and drugs. Consequently, several novel efficient strategies based on catalytic methods have been validated to date for the direct assessment of such scaffolds in an enantiopure fashion [1,2,3]. In this context, spirocyclic oxindole derivatives have appeared as privileged structural motifs being part of a great number of synthetic and natural products displaying remarkable biological activities as well as useful biomedical applications (Figure 1) [4,5,6]. Asymmetric organocatalysis has appeared as an appealing tool in order to prepare such compounds with rich structural diversity and complexity through cascade, tandem, and domino processes [7,8,9]. Indeed, since the beginning of the 21st century, the golden-age of organocatalysis, several reports and substantial advances on the field of the synthesis of complex structural entities via the combination of organocatalysis and cascade transformations have been reported so far. The ability to reach for molecular complexity through the conscious choice of substrates and catalysts in a single transformation has inspired a growing number of research groups. Seminal reports that cover this subject have appeared before 2015 [10,11,12,13].

The aim of this review is to describe recent advances towards the stereocontrolled synthesis of strained spiro-quaternary stereocenters on the oxindole core through non-covalent organocatalysis [14,15,16] within the timeframe from 2015 to the middle of 2017. It is the authors’ aim to attract the reader’s attention to the potential of asymmetric non-covalent organocatalysis to mediate one-pot cascade, tandem, and domino processes that, employing an oxindole derivative as starting material, give a facile access to complex molecules featured by several functionalities and various stereogenic centers.

**Figure 2:**
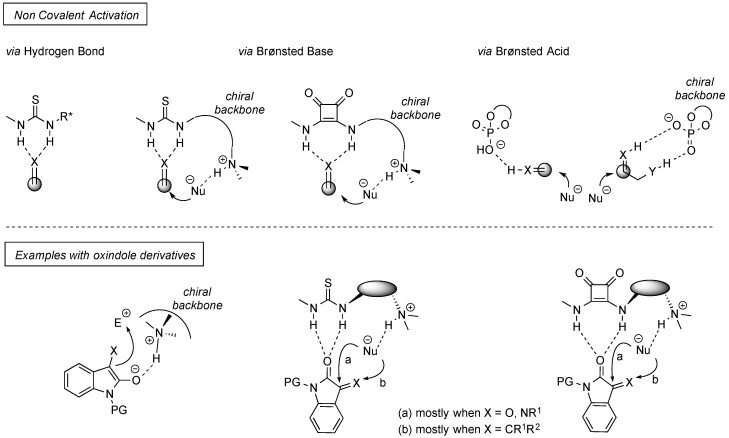
Examples of non-covalent interactions in a substrate/reagent/catalyst system.

The observed high yields and remarkable stereoinduction, which resemble Nature’s outcomes, mainly rely on H-bond interactions, electrostatic effects, and π–π staking that are established between the oxindole core, the employed reagent, and the selected catalyst (i.e., dual and cooperative catalysis) [17]. Examples of such connections are depicted in Figure 2.

Far to be exhaustive and comprehensive, the current review is divided according to the most employed class of catalysts. Specifically, after a quick overview about the *Cinchona* alkaloids (Section 1), the subsequent synthetic elaborations of these natural-occurring organocatalysts are going to be introduced (Section 2, Section 3, Section 4 and Section 5); meanwhile, Section 6 will detail an example of non-covalent activation via Brønsted acids. Finally, we apologize for any omissions.

## 2. Cinchona Alkaloid Catalysts

**Figure 3:**
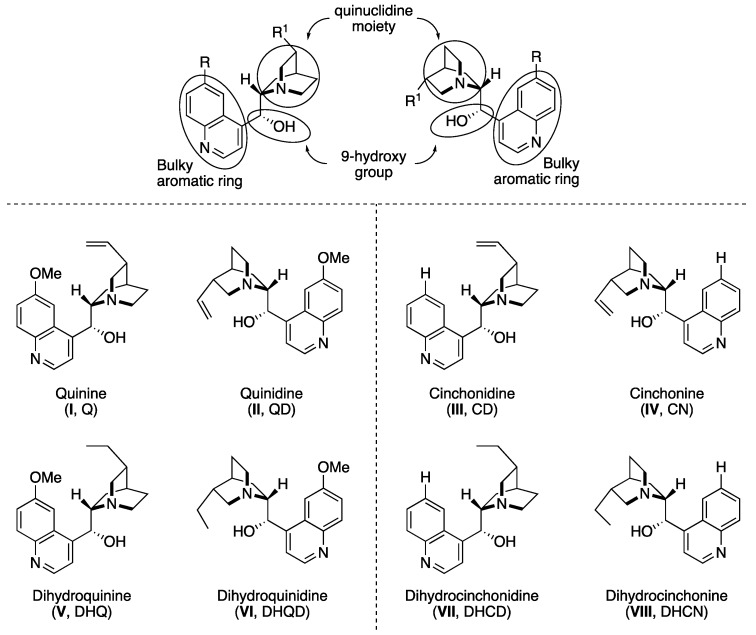
Representative examples of *Cinchona* alkaloids.

Discovered in 1820 for its antimalarial properties, *Cinchona* alkaloids have found several applications in various and quite different fields ranging from medicine to food and beverage industries, and even including organic chemistry. Besides the initial uses as resolving agents, over the last decades, *Cinchona* alkaloids have established a primary and outstanding role in asymmetric synthesis as highly efficient organocatalysts capable of promoting a wide range of enantioselective transformations in both homogeneous and heterogeneous environments [18]. The impressive chiral induction mainly relies both on the accessibility of different chiral skeletons and on the facile adaptability to various reaction processes (Figure 3). Specifically, the 1,2-aminoalcohol group summarizes in one molecule:

*(i)*  the basicity and bulkiness of quinuclidine moiety, apt to activate a nucleophile by deprotonation as well as to stabilize the developing positive charge,
*(ii)*  the secondary 9-hydroxy group, which acts as both an acid and a H-bond donor, and is suitable for further chemical modification of the catalyst structure. Additionally, the overall catalytic action is also ascribed to the possible π–π interactions with the aromatic quinoline ring.

Representative examples of widespread used *Cinchona* alkaloids are depicted in Figure 3. Notably, as fragments of more complex organocatalysts (Section 3, Section 4 and Section 5) the same structures play a crucial role to induce high levels of diastereo- and enantioselectivity.

**Scheme 1:**
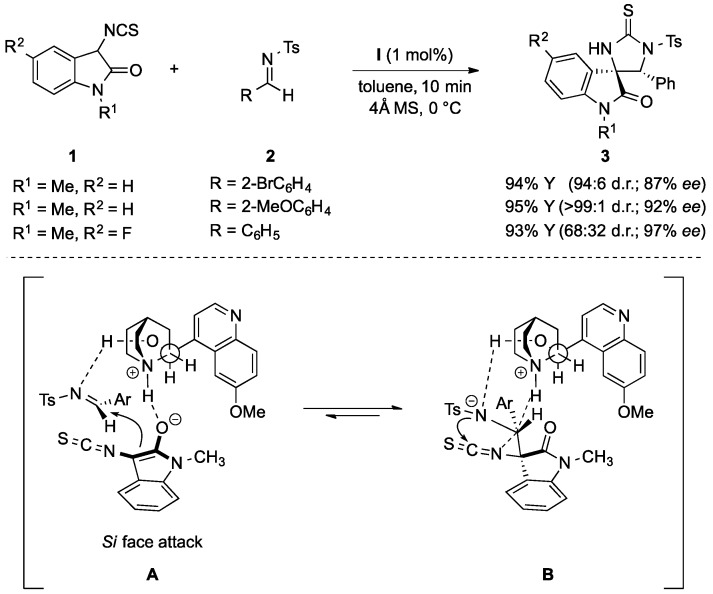
Synthesis of spiro[imidazolidine-2-thione-4,3′-oxindole] via domino Mannich/Cyclization process and proposed transition states where **A** and **B** are the suggested intermediates (Y = Yield).

Within this context, in 2015, Yuan and co-workers succeeded in the construction of a class of spirocyclic oxindoles through a domino Mannich-cyclization process [19]. Specifically, employing various 3-isothiocyanate oxindoles **1** and imines **2** as substrates, the simplest commercially available quinine (**I**, 1 mol %) made possible the diastereo- (up to >99:1 d.r.) and enantioselective (up to 97% *ee*) synthesis of spiro[imidazolidine-2-thione-4,3′-oxindole] derivatives **3** (Scheme 1). Notably, the optimized conditions, i.e., using toluene as solvent at 0 °C with the addition of 4 Å molecular sieves (MS), led, in just 10 min, to the desired products isolated in remarkable yields (up to 95%).

To explain the observed outstanding stereocontrol, the authors suggest that in the transition state the quinine simultaneously activates: (*i*) the tosyl-protected imine via a H-bond interaction of the C9-OH and (*ii*) the 3-isothiocyanate oxindole moiety via deprotonation and consequent enolization performed by the tertiary amine group. In such a way, the imine *Si*-face is exposed to the attack of the *Si*-face nucleophile counterpart (**A**, Scheme 1). The subsequent ring closure reaction involves the just formed *N*-nucleophile and the electron-poor carbon of the isocyanate oxindole framework (**B**, Scheme 1). 

**Scheme 2:**
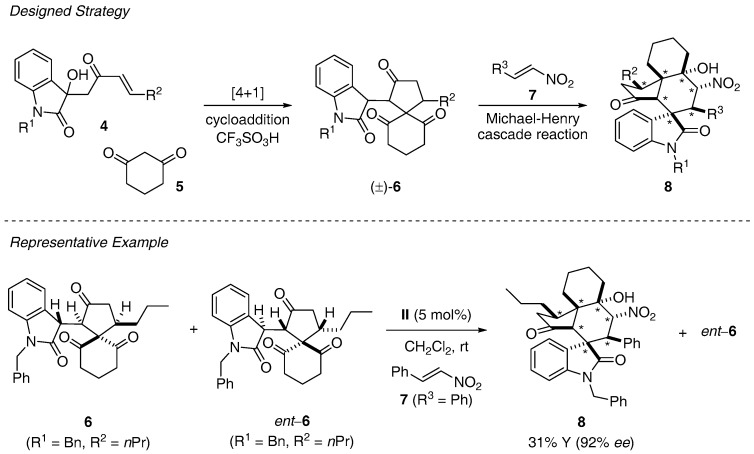
Synthesis of spirooxindole polycycles bearing a spiro[4,5]decane system.

More recently the impressive stereoinduction properties of *Cinchona* alkaloids have been validated even in a kinetic resolution approach. Indeed, Tanaka and co-workers were engaged in the synthesis of spirooxindole polycycles **8** bearing a spiro[4,5]decane system that was remarkably accomplished by a two-step strategy involving a formal [4 + 1] cycloaddition and a subsequent Michael-Henry cascade transformation (Scheme 2) [20].

While the former reaction was carried under acidic catalysis, the Michael-Henry cascade steps were performed under quinidine (**II**) catalysis at room temperature. In such conditions, the final polycyclic derivatives **8** featuring seven stereogenic centers were isolated in high optical purity (80–92% *ee*) despite modest yields (up to 28%) consequence of the racemic mixture used as starting materials. However, the unreacted enantiomer *ent*-**6** was easily recovered in enantioenriched fashion (up to 43% yield, 92–98% *ee*).

Although initially the simplest naturally available *Cinchona* alkaloids have been widely exploited, over the years, organic chemists have created more efficient and complex molecules bearing the same scaffold that was implemented with the introduction of further functionality capable of H-bond interactions. In the following sections, we are going to illustrate and compare other privileged organic chirality inducers, most of which could be added to the realm of *Cinchona* alkaloid derivatives.

## 3. (DHQD)_2_ Based Catalysts

**Scheme 3:**
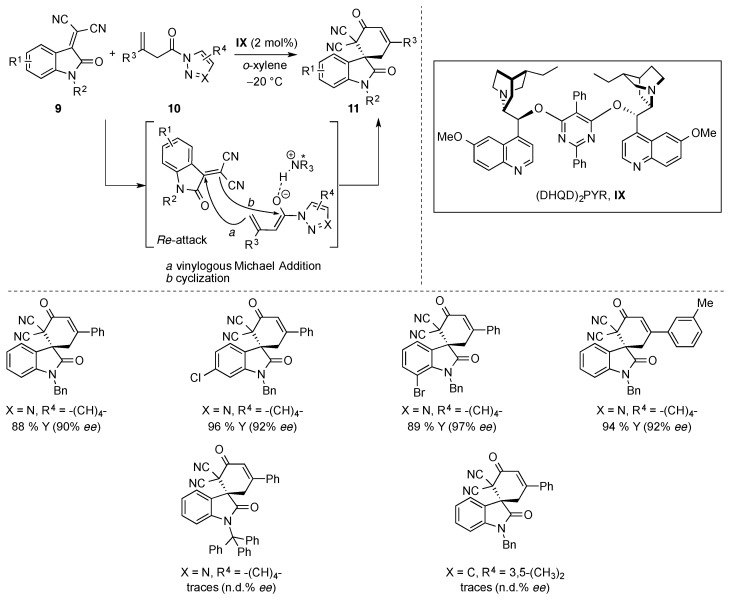
Michael/cyclization cascade reaction to spirociclyc oxindoles catalyzed by (DHQD)_2_PYR (**IX**).

Joining together two units of *Cinchona* derivatives/analogues in a single molecule, creating the so-called *bis*-*Cinchona* alkaloids, is the easiest way to enhance the H-bond network inside the catalyst/substrates system. Such structures have mainly shown their efficiency as chiral ligands in the Sharpless dihydroxylation. However, over the years, several research groups have highlighted their potential and effectiveness as simple organocatalysts without the introduction of further metal salt additives. Among others, Wu and co-workers exploited the ability of (DHQD)_2_PYR (**IX**) in Michael/cyclization cascade reaction to synthesize different spirocyclic oxindoles starting from isatilidene malonitriles as initial electrophile. Precisely, the employment of acyclic β,γ-unsaturated amides **10** as vinylogous enolates, smoothly provided the titled spirooxindoles **11** in good yields (87–95%) and noteworthy enantioselectivity (77–96% *ee*) even though the overall outcomes were affected by the steric hindrance introduced on both donor and acceptor reagents (Scheme 3) [21].

**Scheme 4:**
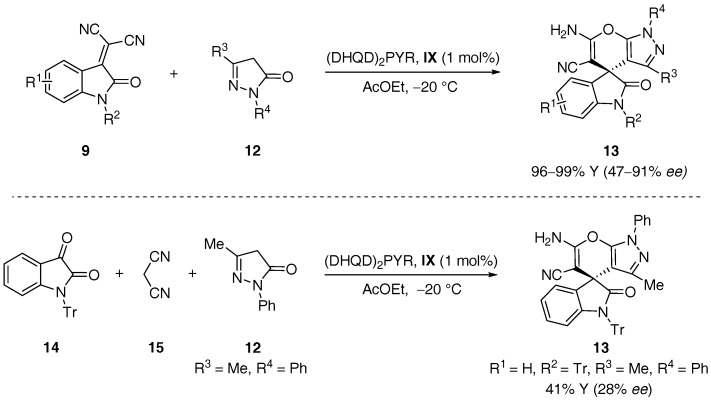
Michael/cyclization cascade reaction catalysed by (DHQD)_2_PYR (**IX**) when pyrazolone **12** was employed as Michael donor.

Likewise, the replacement of the Michael donor with suitable pyrazolone **12** furnished the expected spiro[indoline-3,4′-pyrano[2,3-*c*]pyrazole] derivatives **13** by performing the reaction in analogous conditions (i.e., −20 °C, AcOEt as solvent) and adding only 1 mol % of the chosen organocatalyst **IX** (Scheme 4) [22]. The whole process was completed in a shorter reaction time (from 10 min to 9 h) with respect the previous vinylogous Michael/cyclization sequence (from 60 min to 7 days) and provided the products in notable yields (96–99%) and good-to-moderate optical purity (47–91% *ee*).

Unfortunately, the optimized protocol failed when the one-pot three component reaction of *N*-trityl isatin (**14**), malonitrile (**15**), and pyrazolone **12** (R^3^ = Me, R^4^ = Ph) was attempted affording the hypothesized spirocompound **13** (R^1^ = H, R^2^ = Tr, R^3^ = Me, R^4^ = Ph) in low yield (41%) and poor enantioselectivity (28% *ee*).

**Scheme 5:**
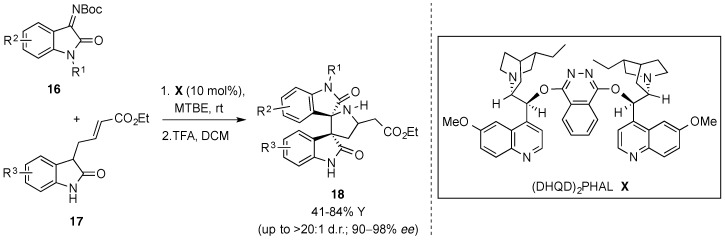
Organocatalytic Mannich/Boc-deprotection/aza-Michael sequence developed by Enders and co-workers.

Almost simultaneously, Enders and co-workers designed and realized an organocatalytic Mannich/Boc-deprotection/aza-Michael sequence of *N*-Boc ketimine **16** and 3-substituted oxindoles **17** that straightforwardly afforded the functionalized 3,3′-pyrrolidinyl derivatives **18** bearing three stereocenters, two of which were contiguous spiro-stereocenters [23]. Therefore, the devised and validated protocol (i.e., room temperature, MTBE as solvent), which relied on the efficiency of (DHQD)_2_PHAL **X** (10 mol %) as catalyst, gave access in moderate-to-good yields (41–84%) and high stereoselectivity (up to >20:1 d.r., 90–98% *ee*) to complex spirocyclic systems **18** (Scheme 5).

## 4. Thiourea-Based Catalysts

**Figure 4:**
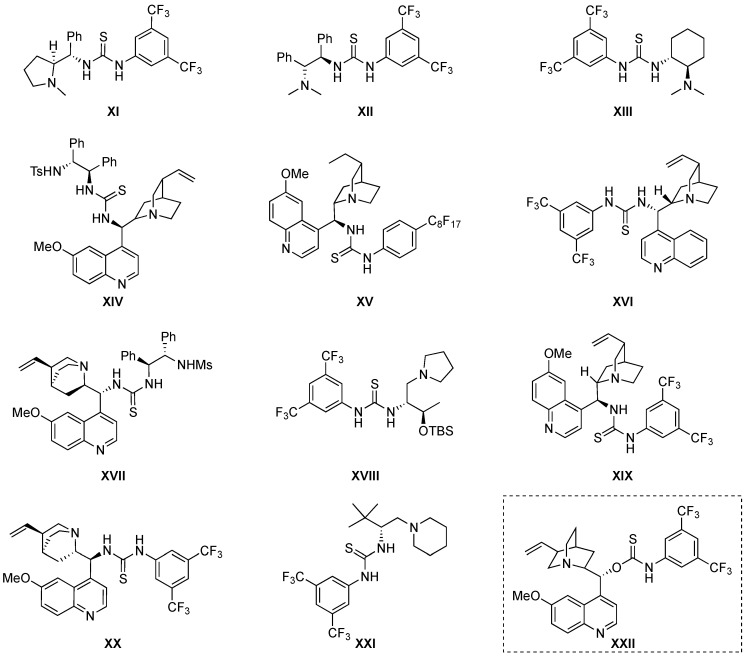
Thiourea and thiocarbamate organocatalysts used in this section.

Thiourea-based organocatalysts [24,25,26,27] have been intensively considered for promoting multiple C-C and C-heteroatom bonds formation via domino reactions through H-bond network between substrates and catalysts. In this section, recent selected cascade reactions involving such organocatalysts (Figure 4) allowing to highly functionalized chiral spirooxindole-bearing compounds are going to be discussed.

### 4.1. Michael Addition/Cyclization Sequence

**Scheme 6:**
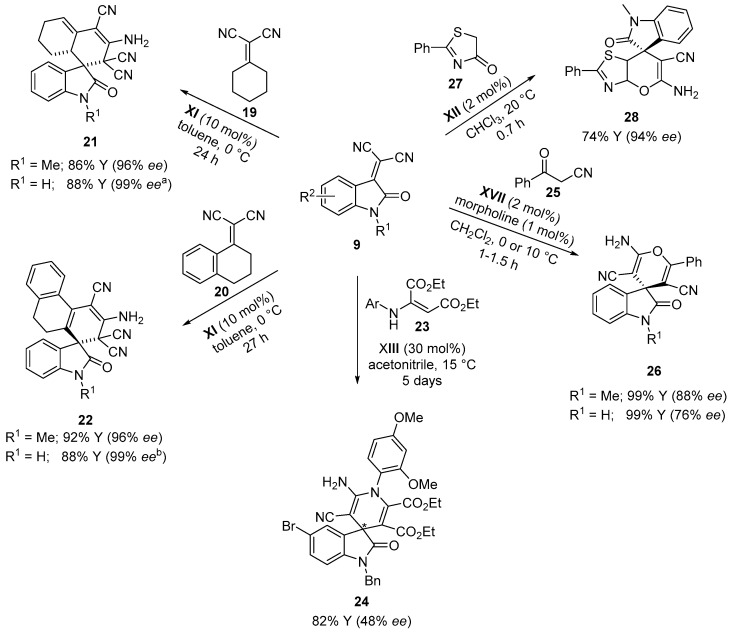
Recent examples of spirooxindoles synthesized from isatylidene malonitrile derivatives **9** through cascade processes involving Michael addition/cyclization sequence. ^a^ Three-component reaction performed during 36 h. ^b^ Three-component reaction performed during 48 h.

Electrophilic isatilydene malonitrile derivatives **9** are substrates of choice to promote Michael addition/cyclization transformations through reaction with nucleophiles. In 2015, Kesavan and co-workers have indeed devised a sequential vinylogous Michael addition/cyclization in the presence of vinyl malononitriles **19** and **20** as vinylogous nucleophiles [28]. The reaction, conducted in the presence of L-proline derived bifunctional thiourea catalyst **XI** (10 mol %) in toluene at 0 °C, demonstrated a wide scope with both substrates (Scheme 6, compounds **21** and **22**). In addition, an enantioselective three-component reaction could also be proposed via in situ formation of isatylidene malonitrile derivatives. The authors have also highlighted the ability of **XI** to afford high levels of enantioselectivity without the need for *N*-protected oxindoles. Indeed, enantioselective transformations involving oxindoles usually require the prior *N*-protection in order to avoid unsought substrate interactions with the catalyst.

Herrera and co-workers reported the combination of enamines **23** and isatylidene malonitrile **9** to propose a promising asymmetric synthesis of few 2-oxospiro-[indole-3,4′-(1′,4′-dihydropyridine)] **24** in moderate yields and enantioselectivities (Scheme 6, compound **24**) [29]. The domino process is believed to follow a mechanism that involves a Michael addition, an intramolecular cyclization and then, a tautomerization and is catalyzed by Takemoto’s catalyst [30] **XIII** (30 mol %) in acetonitrile at 15 °C.

The synthesis of several spiro[4*H*-pyran-oxindole] derivatives **26** was proposed by Wu and co-workers by using α-cyano ketones **25** as nucleophiles (Scheme 6) [31]. In the presence of very low loadings of quinidine-derived thiourea organocatalyst **XVII** (2 mol %) and morpholine (1 mol %) in dichloromethane at 0 or −10 °C, the cascade process takes place within less than two hours and accommodates a broad range of substrates affording the expected products in excellent yields and high enantioselectivities. The authors have shown that the chiral tertiary amine moiety in catalyst **XVII** is crucial to afford enantioenriched spiro compounds **26** as in its absence, the expected products were obtained in a racemic manner.

Novel thiazole-fuzed spirooxindoles **28** were synthesized in high yields and enantioselectivities by using (1*R*,2*R*)-1,2-diphenylethane-1,2-diamine derived thiourea catalyst **XII** (2 mol %) (Scheme 6) [32]. In this case, the domino transformation also takes place in shorter reaction times and the catalytic system proved to be suitable to a series of 2-substituted thiazol-4-ones **27** as nucleophiles and 2-(1-methyl-2-oxoindolin-3-ylidene)malonitrile as electrophile. Concerning the mechanism, the authors reasoned that the observed stereochemistry of this domino reaction can be explained via a first Michael addition of thiazolones **27** to **9** to afford the Michael addition intermediate followed by its subsequent intramolecular Thorpe-Ziegler-type cyclization. Both steps operate through dual activation of substrates in the presence of the bifunctional thiourea catalyst **XII**.

**Scheme 7:**
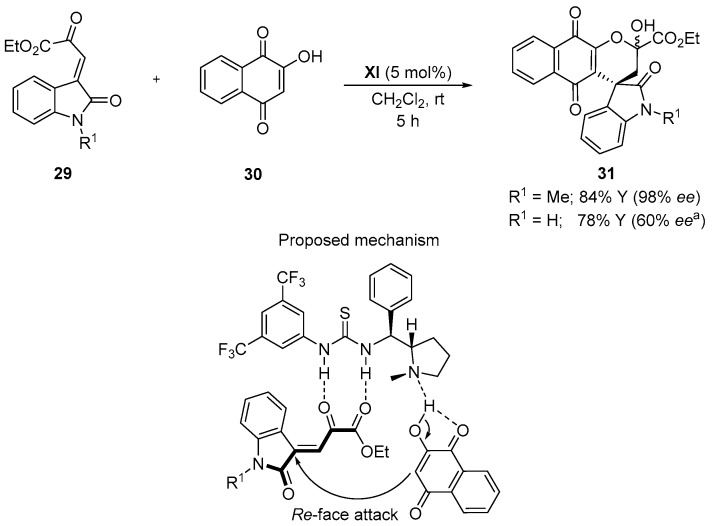
Synthesis of hybrid spirooxindole-naphthoquinones through catalysis with proline-based catalyst **XI**. ^a^ Reaction was carried out at 5 °C.

The combination of naphthoquinone and chromenone derivatives with oxindole ketoesters as Michael acceptors has paved the way to the preparation of versatile heterocyclic compounds. The group of Kesavan has, once again, underscored proline-based catalyst **XI** (5 mol %) as powerful catalyst to carry on tandem Michael addition/hemiketalization of ketoester **29** with 2-hydroxy-1,4-naphthoquinone **30** in dichloromethane at room temperature (Scheme 7) [33]. The expected hybrid spirooxindole-naphthoquinone compounds **31** were obtained in excellent yields and enantioselectivities and displayed good functional tolerance concerning the oxindole ketoester scaffold including different *N*-protecting groups. However, unprotected (i.e., *N*-H) oxindole substrates gave lower yields and enantioselectivities probably due to the additional H-bond binding site that might be in competition either with the organocatalyst or with the substrates. Independently and shortly after, the group of Wang proposed a similar transformation in the presence of catalyst **XX** (10 mol %). In this work, a broader scope has been proposed with respect of both substrates [34]. Additionally, the synthesis of several optically active spiro[oxindole-benzo[*g*]chromene-dione] derivates was also described via cascade reaction between 2-hydroxy-1,4-naphthoquinone as nucleophile and oxindole ketoesters.

**Scheme 8:**
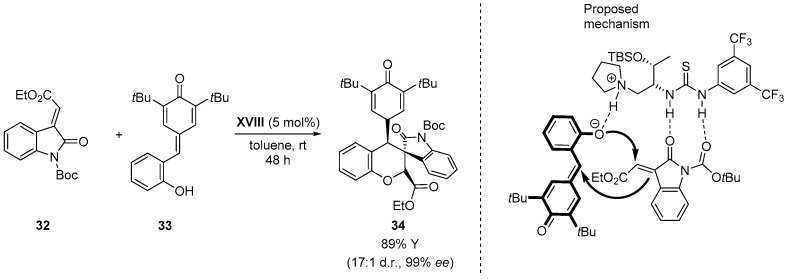
Synthesis of chromans **34** with an oxindole moiety catalyzed by **XVIII**.

In 2016, Enders and co-workers developed a highly selective domino oxa-Michael/1,6-addition sequence to synthesize functionalized chromans with an oxindole moiety [35]. The success of the transformation relies on the use of unprecedented *ortho*-hydroxyphenyl-substituted *para*-quinone methide **33** as donor-Michael acceptor substrates in combination with isatin-derived enoate **32** (Scheme 8). The mild reaction conditions [i.e., catalyst **XVIII** (5 mol %) in toluene at room temperature] showed significantly wide substrate scope and functional group tolerance.

**Scheme 9:**
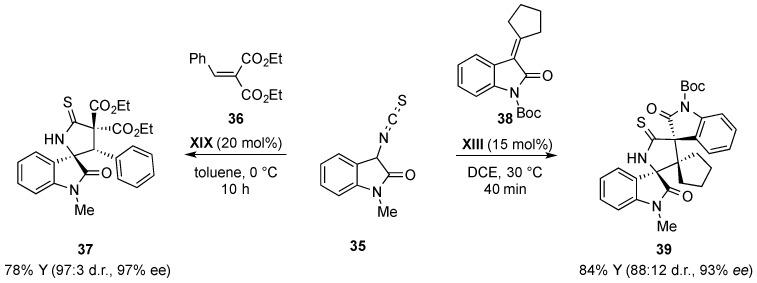
3-Isothiocyanate oxindoles as versatile substrates for the synthesis of densely functionalized spirooxindoles.

Other interesting substrates used in cascade transformations to afford highly substituted and poly-functionalized spirooxindoles through Michael addition/cyclization process are 3-isothiocyanate oxindoles (Scheme 9, compound **35**). Indeed, their ambiphilic character (e.g*.*, bearing both electrophilic and nucleophilic sites) allows for the synthesis of several functionalized spirooxindoles via reaction with diversified suitable substrates such as electron-poor olefins. Chowdhury, Ghosh, and co-workers proposed the use of quinine-derived thiourea catalyst **XIX** (20 mol %) in toluene at 0 °C in order to synthesize a broad range of 3,2′-pyrrolidinyl spirooxindoles **37** in high yields and excellent diastereo- and enantioselectivities by using quite unreactive π-electrophiles such as diethyl benzylidene malonate **36** [36]. Moreover, Jing, Qin, and co-workers reported an asymmetric synthesis of *trans*-configured trispirooxindoles by combining **35** and cyclic methyleneindolinones **38** in the presence of Takemoto’s catalyst **XIII** (15 mol %) [37]. Interestingly, less than 60 min is enough to the cascade reaction to reach completion and the expected spiro compounds that bears three quaternary stereocenters are isolated in good yield and selectivities.

**Scheme 10:**
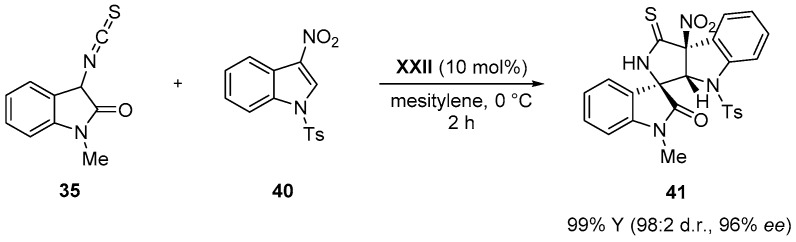
Asymmetric synthesis of polycyclic spirooxindoles in the presence of amino-thiocarbamate catalyst **XXII**.

Remarkably, the use of chiral amino-thiocarbamate catalyst **XXII** (10 mol %) gave rise to several polycyclic spirooxindoles **41** containing three contiguous chiral centers, with two of them having quaternary stereocenters, in the presence of **35** and 3-nitroindoles **40** (Scheme 10) [38]. Excellent yields and selectivitivites were obtained when the N1-position of **35** was blocked with a methyl group while slight erosion of the diastereo- and enantioselectivities were observed for more hindered N1-protecting groups.

### 4.2. Michael Addition/Mannich/Cyclization Sequence

**Scheme 11:**
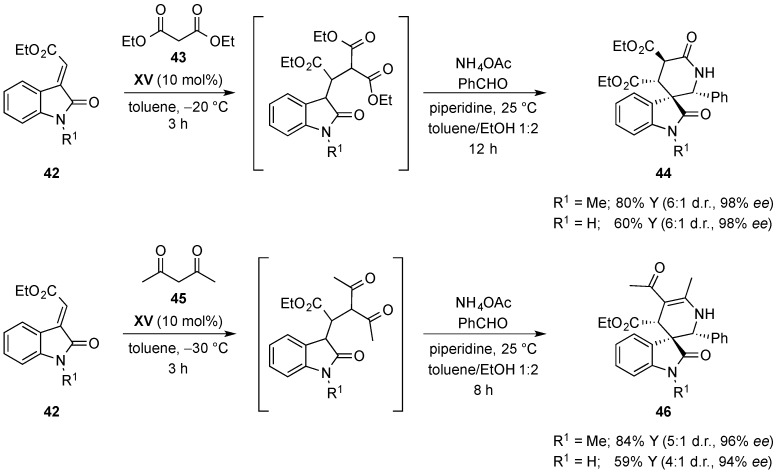
Synthesis of piperidone and tetrahydropyridine rings bearing spirooxindoles in the presence of recyclable catalyst **XV**.

Recyclable fluorous bifunctional *Cinchona* alkaloid catalyst **XV** [39] has proved its efficiency to catalyze cascade reaction in the presence of electron-deficient olefinic oxindole **42** and nucleophiles. The syntheses of spirooxindoles containing 2-piperidinone **44** and tetrahydropyridine **46** rings were successfully accomplished in 2015 by Zhang and co-workers through a four-component cascade transformation in the presence of diethyl malonate **43** or 1,3-diketone **45** respectively (Scheme 11) [40]. Under the optimal conditions [i.e., catalyst **XV** (10 mol %) in toluene] high yields and levels of selectivity were reached affording polycyclic molecules densely functionalized that were prone to either further derivatization or scale-up. Although the authors have mainly proposed the use of oxindoles bearing a methyl group at the N1-position, one single example using free-N1 has been described affording slightly lower yields and comparable selectivities.

**Scheme 12:**
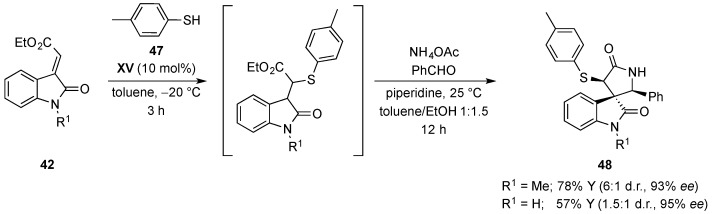
Synthesis of spiro-γ-lactam oxindoles via cascade reaction catalyzed by catalyst **XV**.

Shortly after, the same group has used catalyst **XV** (10 mol %) during a similar cascade reaction for the synthesis of spiro-γ-lactam oxindoles via a thiol-Michael/Mannich/lactamization cascade reaction in good yields and enantioselectivities and moderate diastereoselectivities [41]. The method goes through a four-component/one-pot synthesis and paves the way to novel compounds **48** containing three contiguous stereocenters including a quaternary one (Scheme 12). As for the previous example, *N*-Me indoles were exclusively used as substrates and only one example with *N*-H was reported. In both cases, catalyst recovery has been realized through first (*i*) loading onto a fluorous silica gel cartridge for solid-phase extraction (F-SPE) [42] followed by (*ii*) elution with 80:20 MeOH/H_2_O for products and other non-fluorous components and 100% MeOH for the catalyst **XV**. Overall, catalyst is recovered in more than 91% yield and >97% of optical purity.

**Scheme 13:**
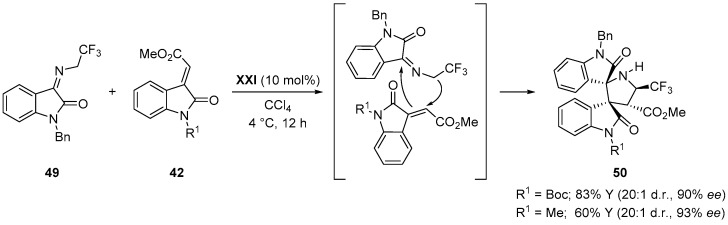
Domino Michael-Mannich [3 + 2] cycloaddition for the asymmetric synthesis of 3,3′-pyrrolidinlyl-dispirooxindoles **50**.

Recently, Enders and co-workers have described the asymmetric preparation of trifluoromethylated 3,3′-pyrrolidinyl-dispirooxindole derivatives bearing four contiguous stereogenic centers among which two are vicinal [43]. The Michael-Mannich [3 + 2] cycloaddition takes place in the presence of oxindoles **42** and **49** through thiourea-based derivative **XXI** catalysis (Scheme 13). Concerning the scope, several olefinic oxindoles **42** (containing electron-neutral, electron-donating or electron-withdrawing groups in the benzene ring) as well as trifluoroethyl isatin ketimines **49** (bearing electron-donating or a 5-F groups in the benzene moiety) are well tolerated. To replace the Boc protecting group by a Me group on the nitrogen of substrate **42** did not hamper the enantioselectivities. Under the optimized conditions (i.e., **XXI** (10 mol %) in CCl_4_ at 4 °C for 12 h) the authors described 17 novel 3,3′-pyrrolidinlyl-dispirooxindoles **50** in good yields (60–92%), moderate-to-good diastereoselectivities (4:1 to >20:1 d.r.) and enantioselectivities (72–93% *ee*). In addition, scale up on a gram scale of the reaction was realized conserving high yields and keeping the same levels of selectivities. 

### 4.3. Double Michael Addition Sequence

**Scheme 14:**
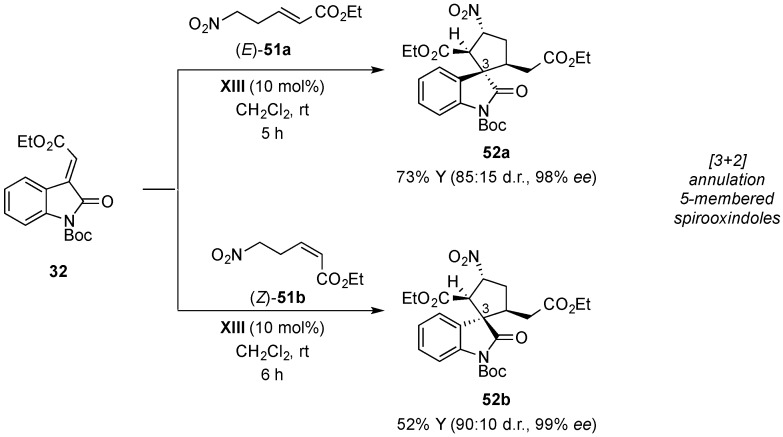
Synthesis of β-nitro spirocyclopentane indolinones **52** via double Michael cascade in the presence of Takemoto’s catalyst **XIII**.

**Scheme 15:**
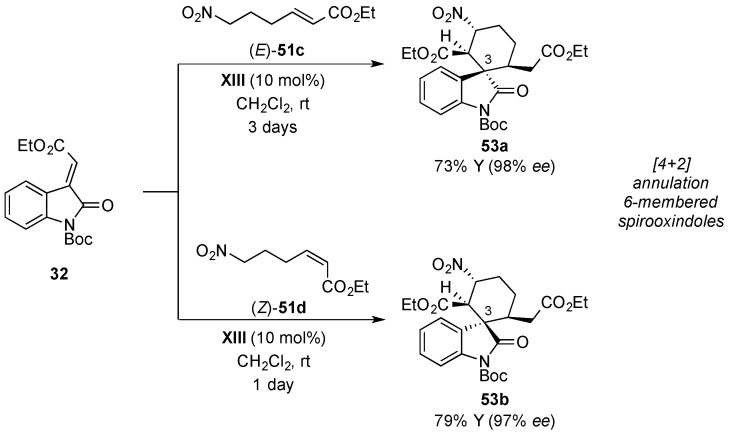
Synthesis of β-nitro spirocyclohexane indolinones **53** via double Michael cascade in the presence of Takemoto’s catalyst **XIII**.

A double Michael cascade reaction sequence, that allowed for a stereoselective [3+2] and [4+2] spiroannulation process, gave rise to both five- and six-membered β-nitro spirocarbocyclic oxindoles respectively [44]. After the screening of several thiourea-based organocatalysts, Quintavalla and co-workers have identified Takemoto’s catalyst **XIII** (10 mol %) as the most effective in terms of yields and selectivities. By combining 2-(2-oxoindolin-3-ylidene)-acetic esters **32** and nitroenoates **51** as donor/acceptor compounds, novel spirooxindoles densely functionalized were isolated via this Michael-Michael cascade process (Scheme 14 and Scheme 15). Noteworthy to mention, the authors foreground that upon varying the double bond geometry (*E* or *Z*) of nitroesters **51**, the configuration of the spiro quaternary stereocenter is inverted affording C3-epimers. This observation is valid for both five-(**52**) and six-membered (**53**) spirooxindoles. If the absolute configuration of the spiro center was determined accordingly to the *E*/*Z* geometry of nitroesters double-bonds, the remaining stereocenters were forged under the catalyst **XIII** control.

### 4.4. Aldol/Lactonization/Elimination Sequence

**Scheme 16:**
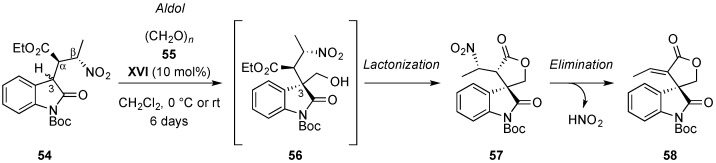
Synthesis of 3-spiro-α-alkylidene-γ-butyromactone oxindoles **58** in the presence of Cinchona-derived thiourea catalyst **XVI**.

An enantioselective domino reaction involving an unprecedented one-pot aldol/lactonization/elimination sequence has been developed, in 2016, yielding a broad range of 3-spiro-α-alkylidene-γ-butyrolactone oxindoles **58** (Scheme 16) [45]. The reaction is performed in the presence of β-nitro indolin-2-ones **54** and paraformaldehyde **55** as starting materials and it is catalysed by bifunctional *Cinchona*-derived thiourea **XVI** (10 mol %) in dichloromethane at 0 °C or room temperature. While **54** was used as a 1:1 mixture of C3 epimers, where both the Cα and Cβ absolute configuration were fixed and known [46], the expected products were isolated with well-established C3 quaternary spirocenter. Even though the two well defined Cα and Cβ stereocenters are destroyed during the domino process, it is interesting to mention that the only stereolabile C3 center of **54** became the unique controlled and defined one present on the final products. It was postulated that the reaction might proceed through an aldol reaction between **54** and **55** to afford the acyclic intermediate **56** which bears three stereodefined centers including the fixed C3 quaternary one. Then, lactonization affords the cyclic lactone **57** which in turn loses its nitro group through HNO_2_ extrusion to afford the expected compounds **58** (Scheme 16).

### 4.5. Friedel-Crafts/Hemiketalization Sequence

**Scheme 17:**
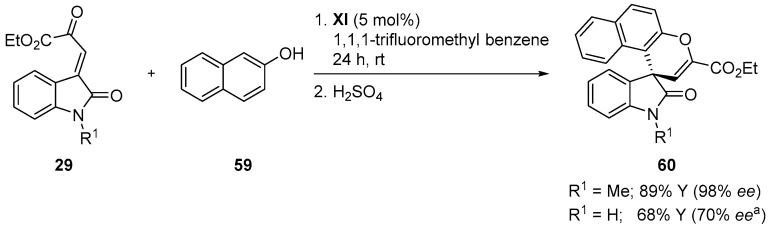
Synthesis of spirooxindole-naphthopyrans **60** in the presence of catalyst **XI**. ^a^ 86% *ee* after recrystallization.

The Kesavan group has published a straightforward synthesis of several oxindole-fused naphthopyran derivatives **60** by combining oxindole α-ketoester **29** and 2-naphthol **59** and using a sequence of Friedel-Crafts-hemiketalization reactions [47]. Under the optimized conditions (i.e*.*, **XI** (5 mol %), in 1,1,1-trifluoromethyl benzene at room temperature) N1-protected oxindoles afforded the expected products in good yields and enantioselectivities. However, unprotected *N*-H oxindoles conducted to lower yields and selectivities probably due to competitive binding of the *N*-H site with the catalyst that might partially hamper the catalytic activity (Scheme 17).

### 4.6. Miscellaneous

**Scheme 18:**
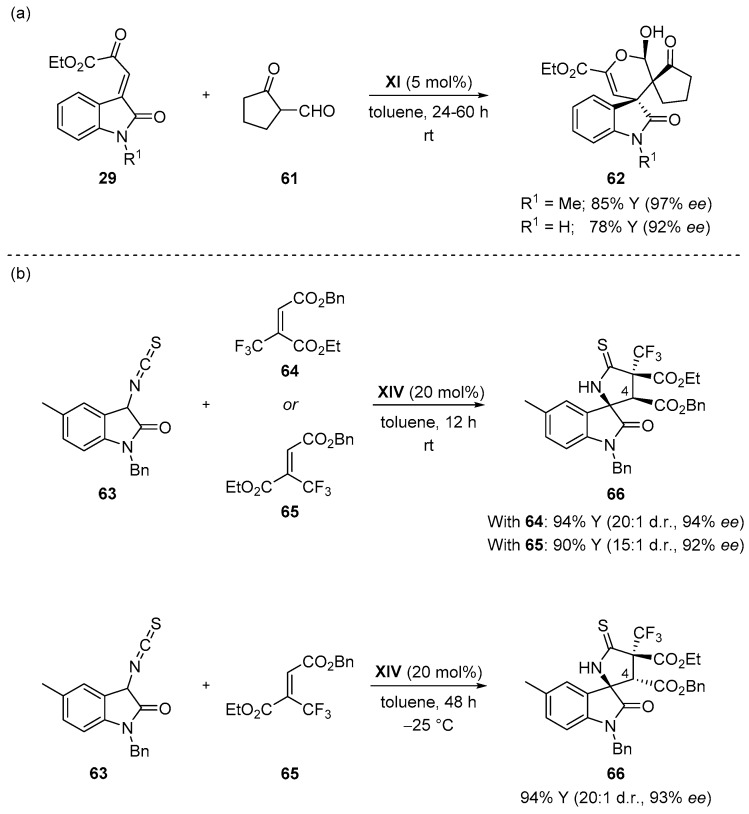
(**a**) Synthesis of spiro-3,4-dihydropyrans **62** and (**b**) synthesis of trifluoromethylated spirooxindoles derivatives **66**.

The synthesis of spiro-3,4-dihydropyrans **62** bearing three stereocenters with vicinal quaternary ones has been reported by Kesavan and co-workers through the use of catalyst **XI** (5 mol %) in toluene at room temperature (Scheme 18a) [48]. Supported by the obtained *syn* configuration of the secondary alcohol and the cyclopentanone moiety, the authors defend an inverse-electron-demand hetero-Diels-Alder reaction pathway of oxindole α-ketoester **29** with cyclic β-oxoaldehyde **61** rather than a cascade transformation via Micheal addition/hemiketalization. An efficient enantioselective [3+2] cyclization of 3-isothiocyanate oxindoles **63** and trifluoromethylated 2-butenedioic acid diester **64** or **65** paved the way to the synthesis of spirooxindoles with a CF_3_-containing all-carbon stereogenic center **66** (Scheme 18b) [49]. Interestingly, the authors highlighted the possibility to obtain either isomers (i.e., epimers at C4 position) in the presence of the same catalyst **XIV** (20 mol %) at different temperatures. This observation relies on the ability of isomerization of dimethyl maleate **64** into dimethyl fumarate **65** through azomethine ylide intermediate in the presence of the amine moiety of aminals [50].

## 5. Squaramide Catalysts

**Figure 5:**
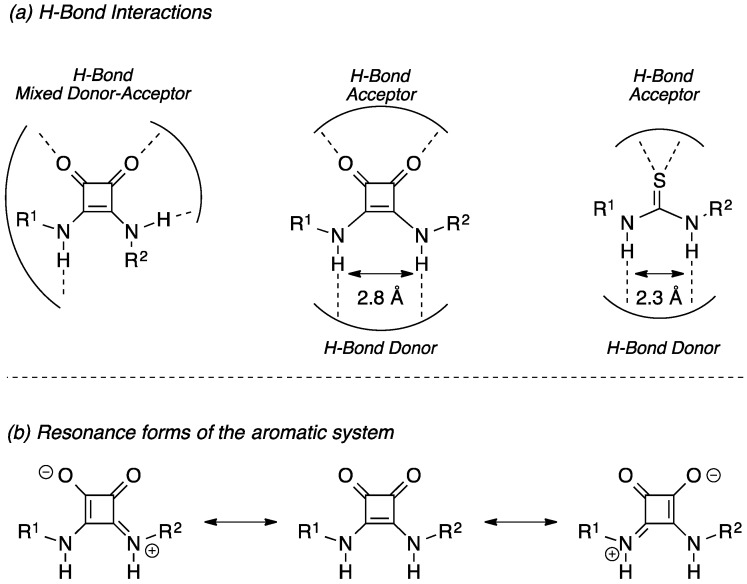
(**a**) H-Bond donor ability of squaramide and thiourea; (**b**) Lone pairs conjugation and resonance forms of the aromatic system.

Inspired by the ability of urea/thiourea derivatives to promote high stereoselective organocatalytic reactions, several research groups have been engaged in the construction of alternative and complementary catalysts that involve in their architecture less explored H-bond donor motifs. Among others, in 2008 Rawal and co-workers postulated the potential of squaramide catalophores [51]. Specifically, over the years, several thorough studies have recognized that secondary squaramides featured by two H-bond donors (NH) and two H-bond carbonyl acceptors (C=O) should easily establish a strong hydrogen bond network with acceptors and donors as well as with mixed acceptor-donor systems (Figure 5a).

Additionally: (*i*) the conformational restriction due to the aromaticity enhancement of the cyclobutendione core upon the delocalization of nitrogen lone pairs, which make the two N-H bonds coplanar with the rigid “squara structure” and (*ii*) the distance between the two N-H that is of 0.6 Å broader than in thioureas, make squaramide derivatives not only a valuable alternative to the urea/thiourea counterpart but also a class of more wide-range applicable organocatalysts (Figure 5b).

**Figure 6:**
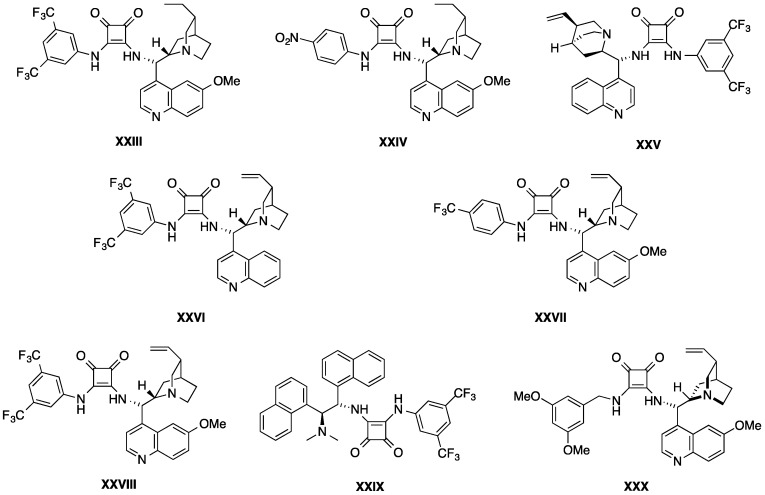
Squaramide based organocatalysts reported in the present section.

Herein we are going to depict selected recent examples that demonstrate their remarkable ability in various cascade reactions involving an oxindole derivative as substrate and the catalophores depicted below (Figure 6).

### 5.1. Double Michael Addition Sequence

**Scheme 19:**
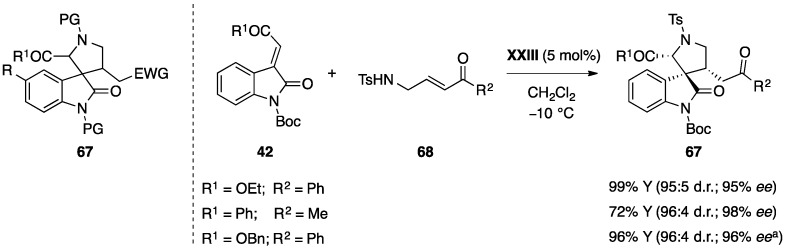
Asymmetric cascade aza-Michael/Michael addition for spiro[pyrrolidine-3,3′-oxindoles]s **67**. ^a^ The reaction was carried out on gram scale.

As stated before, oxindole derivatives have been a privileged substrate in organocatalysis. Specifically, as consequence of its unique electron-demand, α-alkylidene oxindoles have been often involved in cascade reactions mainly relying on Michael addition promoted by both C-nucleophiles and hetero atoms. In such a scenario, squaramide derivatives clearly have ever played a predominant role since their undiscussed ability as bifunctional catalysts [52]. Notably in this field Zhao and Du devoted their efforts not only to the design and synthesis of novel more efficient squaramide organocatalysts but also to the preparation of uncommon cascade reagents that should be suitable to build quite complex and densely functionalized carbocycles. Thus, in 2015, their group reported the first squaramide asymmetric protocol for the synthesis of chiral spiro[pyrrolidine-3,3′-oxindole]s **67** (Scheme 19). The optimized cascade aza-Michael/Michael addition sequence, distinguished for its mild conditions (i.e., −10 °C in CH_2_Cl_2_) and quite low catalyst loading (5 mol %), was easily applied to a broad substrate scope achieving the desired products **67** in high yields with excellent diastereo- (up to >99:1 d.r.) and enantioselectivities (from 93% *ee* to >99% *ee* in almost all cases).

**Scheme 20:**
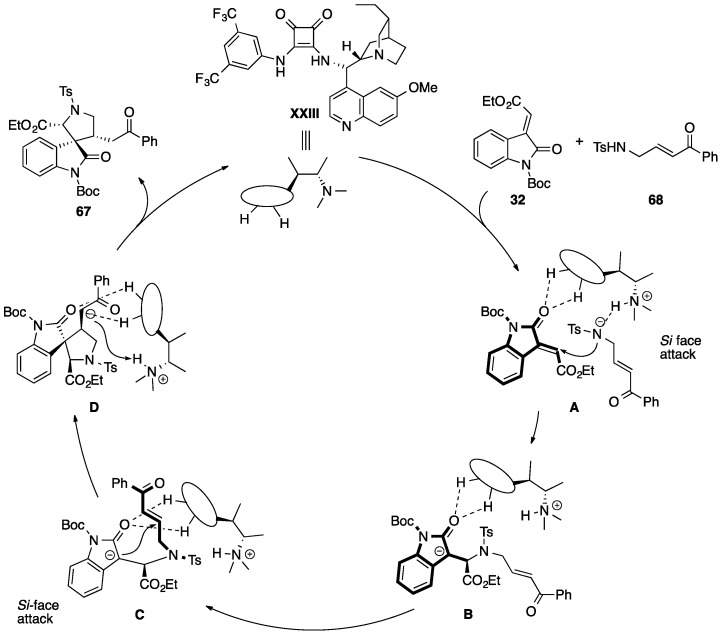
Proposed reaction mechanism for the aza-Michael/Michael cascade reaction developed by Zhao and Du. **A**–**D** are hypothesized intermediates on the catalytic proposed cycle

Remarkably, the reaction could be repeated even on gram scale without any loss in both yield or stereoselectivity. The authors reasoned that such an outstanding stereocontrol should be ascribed to the squaramide moiety that contemporarily activates and orients the (*E*)-*tert*-butyl 3-(2-ethoxy-2-oxoethylidene)-2-oxoindoline-1-carboxylate **32** as well as the hydroquinine framework and enhances the nucleophilicity of the tosylaminomethyl enone **68** (Scheme 20). In the aza-Michael addition, the settled H-bond network (**A**) drives the nitrogen attack only from the *Si* face of the substrate to furnish the intermediate **B** where the enone group undergoes an intramolecular Michael addition on the *Si* face via the transition state **C**, which rapidly provides the product **67** and restores the catalyst **XXIII**.

**Scheme 21:**
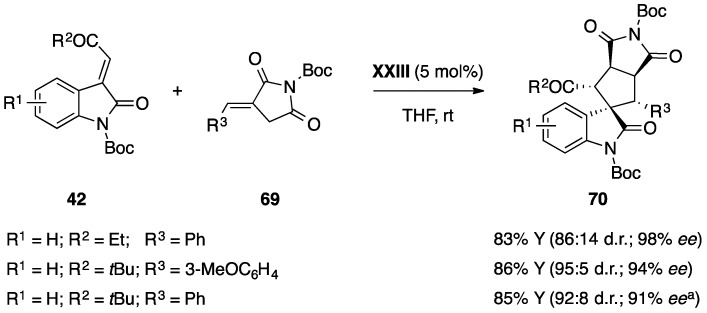
Organocatalytic cascade Michael/Michael addition for spirooxindoles containing five contiguous stereocenter. ^a^ The reaction was carried out on gram scale.

The just disclosed reaction mechanism and the consequent stereochemical outcomes prompted the authors to replace the α,β-unsaturated esters with α-alkylidene succinimides **69** structurally similar to the oxindole counterpart [53]. The previously developed squaramide-catalysed approach in even milder conditions (i.e., room temperature and THF as solvent) guaranteed the formation of highly functionalized spirooxindoles **70** in elevated yield and stereocontrol (up to 97:3 d.r., up to 98% *ee*). In Scheme 21 it is reported the most representative examples of the produced library, which was also synthesized in gram scale without any loss in term of stereoinduction.

**Scheme 22:**
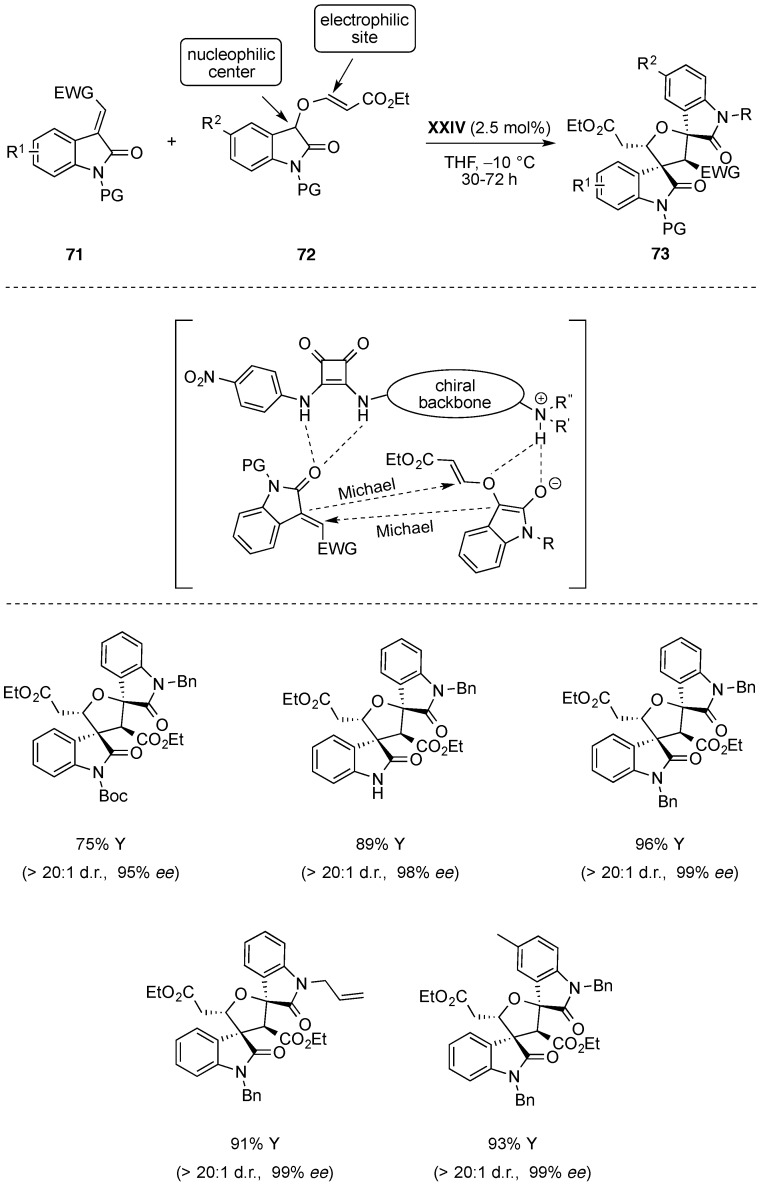
Asymmetric enantioselective tandem Michael/Michael reaction to bispirooxindoles **73**.

Once validated the efficiency of their squaramide **XXIV** as catalyst, in 2016 Zhao and Du designed the new cascade reagent **72** featured by both an active nucleophile center and an electrophile site (Scheme 22) [54]. The authors demonstrated their hypothesis fruitfully employing the developed donor Michael acceptor reagent **72** in a tandem Michael/Michael reaction which smoothly furnished highly functionalized bispirooxindole-tetrahydrofurane scaffolds **73** bearing four contiguous stereocenters. Despite the number of different substituents introduced on each oxindole framework, the overall process never failed to give the expected products **73** in very high levels of optical purity (>20:1 d.r., up to >99% ee) and moderate-to-high yields (58–96% yield).

### 5.2. Michael Addition/Cyclization Reaction Sequence 

A second highly exploited reaction sequence that straightforwardly furnishes complex spiro compounds involves a Michael addition followed by a fast cyclization reaction (see also Section 4.1). Among all the possible substrate/reagent systems largely reported in literature, Yuan and co-workers chose the previously investigated 3-hydroxyoxindoles **75** and 3-aminooxindoles **76** as initial Michael donor and the α,β-unsaturated acylphosphonates as Michael acceptor [55]. Such an uncommon electron poor counterpart was mainly selected due to the lability of the C-P bond that simplifies the phosphonate group removal. Relying on the efficiency of squaramide organocatalyst **XXV** together with the designed starting molecules, the authors developed a highly stereoselective Michael/cyclization cascade sequence able to afford a broad spectrum of spirocyclic oxindoles **78** and **79**. Indeed, applying the initial optimized conditions (i.e., CH_3_CN or CH_2_Cl_2_ as solvent, room temperature), the overall reaction proceeded without any loss in stereoslectivity (up to >99:1 d.r.; 71–97% *ee*) and yield (up to 98%) when (*i*) the Michael donor was either a 3-hydroxy or a 3-aminooxindoles (**75** and **76** respectively), which afford to the spiro γ-lactones (**78** for X = O) and γ-lactames (**79** for X = N), as well as (*ii*) substituents quite different in terms of electron demand and steric hindrance were introduced on both nitrogen and aromatic ring. 

**Scheme 23:**
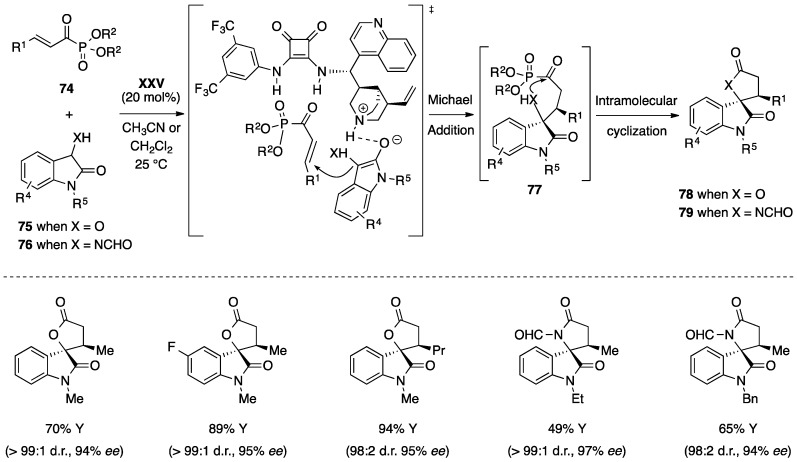
Organocatalysed Michael/cyclization cascade reaction for the construction of spirocyclic oxindole-γ-lactones/lactams.

A slight drop in the stereoinduction was observed when the β position of the α,β-unsaturated acylphosphonate **74** was decorated with excessively bulky groups (Scheme 23). The observed efficiency should be due to the dual activation of the catalyst that (*i*) promotes the enolyzation of the 3-hydroxyoxindoles and (*ii*) exposes the *Re* face of the α,β-unsaturated acyl phosphonate to the attack from the *Si*-face of the just generated nucleophile.

**Scheme 24:**
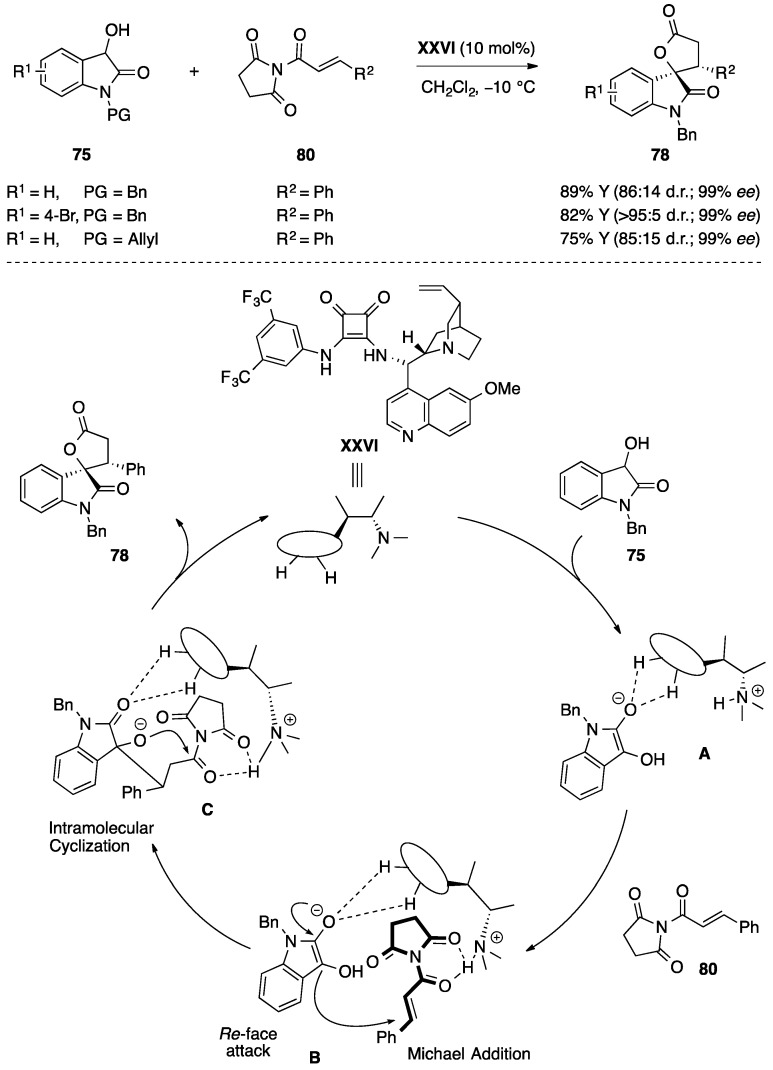
Asymmetric cascade Michael/cyclization reaction performed on 3-hydroxyoxindoles/*N*-acylated succinimides as donor/acceptor system. **A**–**C** are hypothesized intermediates on the catalytic cycle proposed.

More recently, the similar squaramide-quinine based catalyst **XXVI** (10 mol %) was effective when α,β-unsaturated *N*-acylated succinimides **80** were chosen as the initial Michael acceptor and 3-hydroxyoxindoles **75** as the nucleophilic counterpart (Scheme 24). [56] Specifically, after having carefully optimized the reaction conditions (i.e., CH_2_Cl_2_ as solvent, −10 °C as best temperature), Du and co-workers validated their protocol by performing the proposed asymmetric cascade Michael/cyclization reaction on various Michael donor/acceptor systems obtaining the corresponding spirooxindoles lactones **78** in good yields (75–89%). Although the stereochemical outcomes were always excellent in term of enantioselectivity (96–99% *ee*), the diastereomeric ratios seemed to be more easily affected by various substituents introduced both on the enone system and on the oxindole moiety (from 75:25 to >95:25 d.r.). Afterwards, once assigned the correct configuration to the generated stereocenter, the authors provided a mechanistic study that pointed out how the quinine-derived squaramide **XXVI** should act as bifunctional catalyst. As depicted in Scheme 24, the tertiary amine unit deprotonates the 3-hydroxyoxindole while the squaramide moiety binds the resulting nucleophile (**A**) which in turn, attacks the electrophile already activated by the protonated amine (**B**). The Michael adduct **C** undergoes an intramolecular cyclization which removes the succinimide auxiliary and generates the expected spirooxindole **78**.

**Scheme 25:**
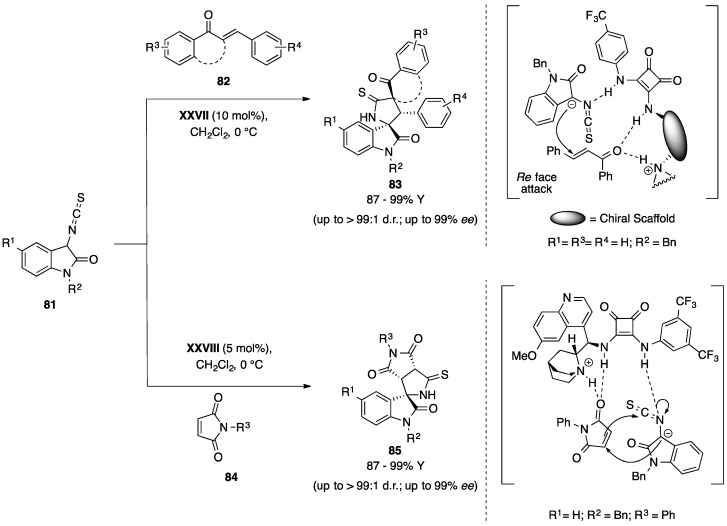
Du’s research toward the preparation of pyrrolidinyl spirooxindoles **83** and **85** employing respectively chalcones **82** and maleimides **84**.

Exploiting the well-known reactivity of 3-isothiocyano oxindoles conferred by the strongly electron withdrawing NCS group, Du and co-workers reported also the preparation of more complex pyrrolidinyl spirooxindoles employing either chalcones **82** [57] or maleimides **84** [58] as acceptor counterpart (Scheme 25). In both cases the extremely similar quinine-derived squaramide **XXVII** (10 mol %) and **XXVIII** (5 mol %) resulted to be the best catalysts in the same reaction condition (i.e., CH_2_Cl_2_, 0 °C). The desired spirocyclic products **83** and **85** were achieved in high yields (87-99%) and excellent diastereo- (up to >99:1 d.r.) and enantioselectivities (up to 99% *ee*) triggered by a complex H-bond network where the organocatalyst enable the stereocontrolled attack of the oxindole donors toward either the electron-poor chalcones **82** or maleimides **84**.

**Scheme 26:**
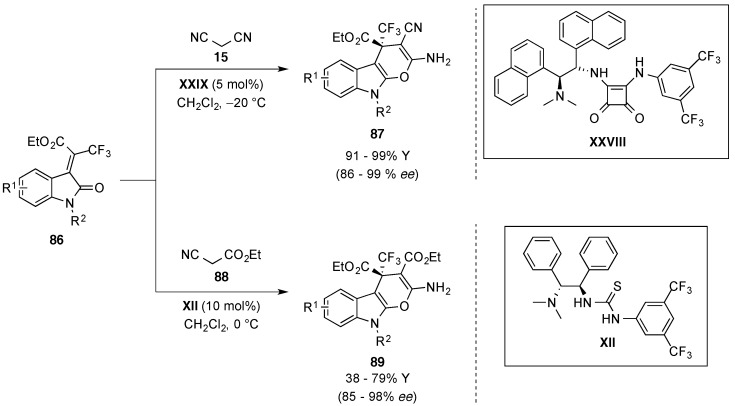
Synthesis of dihydropyranoindole derivatives, a comparison between squaramide-based and thiourea catalyst.

The substitution of quinine moiety with a tertiary ethylenediamine framework decorated with naphthyl groups opened the door to the synthesis of pharmacologically interesting dihydropyranoindole derivatives. Indeed, Zhao and co-workers performed a Michael addition/cyclization sequence at relatively low temperature (−20 °C) in CH_2_Cl_2_ employing the squaramide derivative **XXIX** as organocatalyst able to join together the isatine-based trifluoroacrylates **86** and the malonitrile **15** (Scheme 26) [59]. The hypothesized products **87**, bearing a novel trifluoromethylated all-carbon-substituted stereocenter, were almost always isolated with excellent yields (91–99%) and high level of optical purity (86–99% *ee*). Unfortunately, the optimized protocol failed not only when less hindered substituents (R^2^ = Me, Ac) were introduced as protecting groups in the oxindole unit, but also when the malonitrile **15** was replaced with the less reactive ethyl cyanoacetate **88**. Noteworthy, in the latter case, nearly analogous outcomes in term of yields (38–79%) and stereocontrol (85–98% *ee*) were accomplished only when the squaramide catalyst **XXIX** was substituted with the (1*R*, 2*R*)-diphenyl-1,2-diamine derived bifunctional thiourea **XII**.

The just depicted results, one more time, confirm the complementary use of thiourea-based and squarate derived organocatalyst for the stereoselective preparation of complex bioactive products.

### 5.3. Miscellaneous

**Scheme 27:**
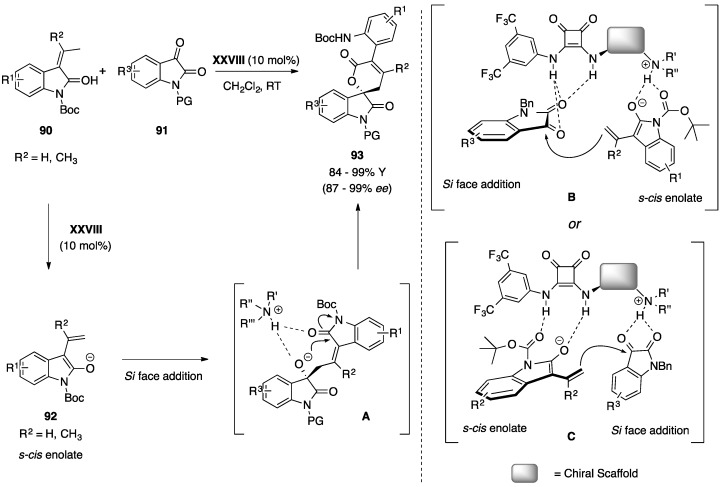
Asymmetric aldol/lactonization sequence for the synthesis of spirooxindole dihydropyranones **93**. **A** is the hypothesized intermediate on the reaction pathway, while **B** and **C** are proposed alternative models for explaining the dual activation of nucleophile and electrophile.

In 2012 Casiraghi and co-workers illustrated how 3-alkylidene oxindoles could also react as vinylogous nucleophile in order to functionalize the γ-position in the presence of a suitable electrophile [60,61]. Inspired by this evidence as well as by subsequent Casiraghi’s researches concerning the Mukaiyama type aldol reactions [62], Han and Chang postulated the creation of biologically relevant 3-hydroxyoxindole framework relying on the nucleophilicity of 3-alkylidenes **90** and the undeniable electrophilicity of isatins **91** [63]. Surprisingly, instead of obtaining the expected aldol adduct, the spirooxindole dihydropyranones **93** were isolated by using the chiral *Cinchona* alkaloid-squaramide bifunctional organocatalyst system **XXVIII** (Scheme 27). The stereochemical outcome of the overall reaction was not influenced by the introduction of different substituents (i.e., in terms of either electron demand or steric hindrance) on both substrates (87–99% *ee*). A slight drop down was observed in the yield only when a NO_2_ group decorated the electrophilic aromatic ring (R^3^ = NO_2_). Without going in fine details, the authors suggested two models for explaining the dual activation of both nucleophile and electrophile (Scheme 27, **B** and **C**) which could account for the *Si* face addition of the s *cis*-enolate in the initial aldol reaction. Once obtained the intermediate **A**, the surprising replacement of the lactam C-N bond with a lactone C-O bond occurs delivering the product **93** after protonation and catalyst regeneration.

**Scheme 28:**
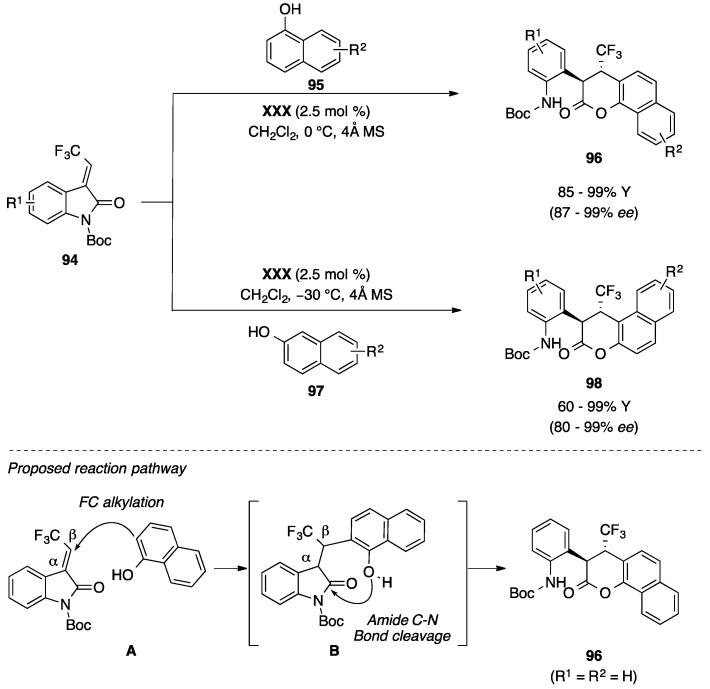
Friedel-Crafts/lactonization domino reaction developed by Zhao and co-workers and suggested reaction pathway.

Such an uncommon behaviour of amide C-N bond was also recently detected by Zhao and co-workers during their studies concerning the organocatalytic Friedel-Crafts/lactonization domino reaction (Scheme 28) [64]. Actually, the authors reported a remarkable example of the employment of squaramide catalophore **XXX** in the asymmetric synthesis of dihydrocoumarins, a ubiquitous scaffold in bioactive natural products. Specifically, the formal [3+3] protocol involved an initial Friedel-Crafts alkylation of naphthols at the C_β_ position of the 3-ylidene oxindoles **94** (**A**, Scheme 28) followed by an unexpected intramolecular cyclization with a C-N bond cleavage of the lactam moiety by the phenolic hydroxyl group (**B**, Scheme 28).

Although the lactonization occurred to disadvantage of the chemically inert oxindole unit, the overall process proceeded under very mild conditions (i.e., CH_2_Cl_2_ at low temperature) and affording attractive outcomes in term of yields (60–99%) and stereoselectivity (>20:1 d.r., 80–98% *ee*) when both α- and β-naphthols (**95** and **97**) were alternatively used as nucleophiles (Scheme 28).

**Scheme 29:**
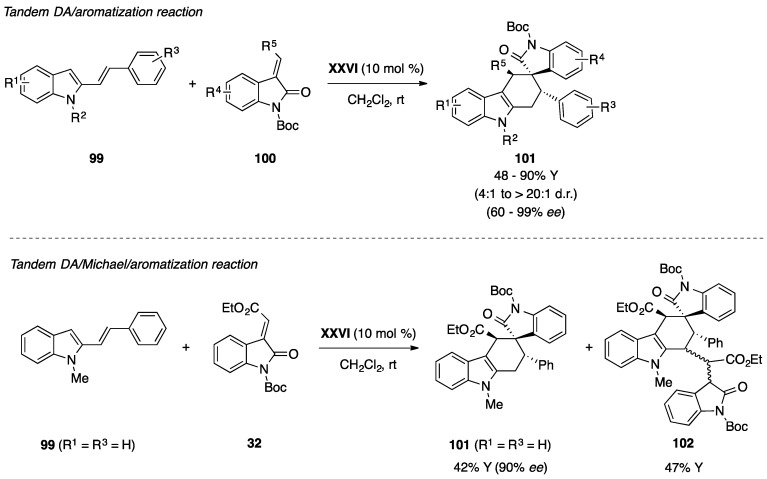
Diels-Alder (DA)/aromatization sequence and triple DA/Michael/aromatization domino process.

*Cinchona*-derived squaramides have shown their potential as organocatalysts even when they are involved in more complex domino reactions. An interesting example was reported by Lu and co-workers in 2015 who initially optimize a simple Diels-Alder/aromatization sequence and then, by adding an excess of oxindole moiety, observed a Diels-Alder/Michael/aromatization domino process (Scheme 29) [65]. The reactions always proceeded smoothly under very mild conditions (i.e., CH_2_Cl_2_ at room temperature) and with quite low catalyst loading (10 mol % of **XXVI**) providing the expected carbazolespirooxindole derivatives **101** in moderate-to-good yields (48–90%) and interesting stereoinduction (from 4:1 to >20:1 d.r., 60–99% *ee*). Unfortunately, for the product **102** with six contiguous stereocenters furnished by the triple domino reaction, it was not possible to define the stereochemical outcome mainly due to the arduous separation of the complex diastereomeric mixture.

## 6. Miscellaneous

**Scheme 30:**
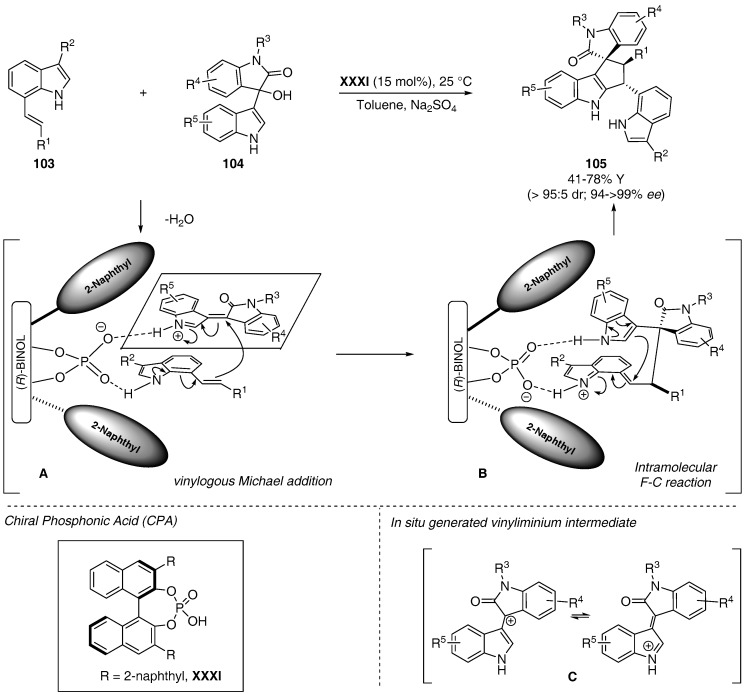
(CPA)-catalyzed Michael addition/intramolecular Friedel-Crafts cascade reaction toward the construction of cyclopenta[b]indole and spirooxindole scaffold. **A**–**C** are postulated intermediates on the mechanism of the reaction.

The impressive reactivity of oxindole derivatives has found several notable applications even when phosphoric acids, a less explored class of catalophores are employed as Brønsted acid organocatalyst. Among the very few reported examples, Shi and co-workers rationally designed a chiral phosphoric acid (CPA)-catalyzed Michael addition/intramolecular Friedel-Crafts cascade reaction toward the construction of cyclopenta[*b*]indole and spirooxindole frameworks [66]. Specifically, the authors supposed that, in order to promote the initial vinylogous Michael addition, the CPA **XXXI** could simultaneously activate throughout a complex H-bond network both the 7-vinylindoles **103** as nucleophile and the electrophilic vinyliminium **C**, which should be generated in situ form the 3-indolylmethanols **104**. Subsequently the transient adduct **B** should undergo, always assisted by the dual H-bond activation of CPA **XXXI**, the intramolecular Friedel-Crafts reaction to restore the initial aromatic indolic structure and furnish the expected complex spirooxindole systems **105** (Scheme 30).

The authors demonstrated their hypothesis successfully performing the diastereo- and enantioselective synthesis of several cyclopenta[*b*]indole derivatives **105** starting from a series of 7-vinylindoles **103** and various isatin-derived 3-indolylmethanols **104**. Comparing the obtained results, it was clear that the stereoinduction was essentially not affected by the different nature of the substituents on both acceptor and donor partners. Conversely, they dramatically influenced the overall reactivity. Particularly, electron-donating groups on C7-functionalised indoles gave much higher yields than the electron-withdrawing groups, while it was less explainable the electronic effect due to substituents on the isatin units.

## 7. Conclusions

As shown by the examples depicted here, when newly developed organocatalysts meet oxindole derivatives, one of the most widespread frameworks in Nature, it is possible to achieve impressive levels of molecular complexity featured by the formation of multiple stereogenic centers upon multiple cascade reactions. Such outstanding results and all the future predictable improvements not only are going to constantly provide easier and easier access to a broad spectrum of highly valuable complex compounds, but also to furnish a better understanding of Nature’s modes of action and ultimately even reach an almost similar degree of perfection.

## References

[1] Wang Y., Lu H., Xu P.F. (2015). Asymmetric catalytic cascade reactions for constructing diverse scaffolds and complex molecules. Acc. Chem. Res..

[2] Cao Z.-Y., Zhou J. (2015). Catalytic asymmetric synthesis of polysubstituted spirocyclopropyl oxindoles: Organocatalysis versus transition metal catalysis. Org. Chem. Front..

[3] Trost B., Brennan M. (2009). Asymmetric syntheses of oxindole and indole spirocyclic alkaloid natural products. Synthesis.

[4] Antonchick A.P., Gerding-Reimers C., Catarinella M., Schurmann M., Preut H., Ziegler S., Rauh D., Waldmann H. (2010). Highly enantioselective synthesis and cellular evaluation of spirooxindoles inspired by natural products. Nat. Chem..

[5] Yu B., Xing H., Yu D.Q., Liu H.M. (2016). Catalytic asymmetric synthesis of biologically important 3-hydroxyoxindoles: An update. Beilstein J. Org. Chem..

[6] Kaur J., Chimni S.S., Mahajan S., Kumar A. (2015). Stereoselective synthesis of 3-amino-2-oxindoles from isatin imines: New scaffolds for bioactivity evaluation. RSC Adv..

[7] Tietze L.F., Brasche G., Gericke K.M. (2006). Domino Reaction in Organic Synthesis.

[8] Volla C.M., Atodiresei I., Rueping M. (2014). Catalytic c-c bond-forming multi-component cascade or domino reactions: Pushing the boundaries of complexity in asymmetric organocatalysis. Chem. Rev..

[9] Grondal C., Jeanty M., Enders D. (2010). Organocatalytic cascade reactions as a new tool in total synthesis. Nat. Chem..

[10] Tian L., Luo Y.-C., Hu X.-Q., Xu P.-F. (2016). Recent developments in the synthesis of chiral compounds with quaternary centers by organocatalytic cascade reactions. Asian J. Org. Chem..

[11] Gasperi T., Vetica F., de Figueiredo R., Orsini M., Tofani D. (2015). Recent advances in organocatalytic cascade reactions toward the formation of quaternary stereocenters. Synthesis.

[12] Hong L., Wang R. (2013). Recent advances in asymmetric organocatalytic construction of 3,3′-spirocyclic oxindoles. Adv. Synth. Catal..

[13] Bencivenni G., Wu L.Y., Mazzanti A., Giannichi B., Pesciaioli F., Song M.P., Bartoli G., Melchiorre P. (2009). Targeting structural and stereochemical complexity by organocascade catalysis: Construction of spirocyclic oxindoles having multiple stereocenters. Angew. Chem. Int. Ed..

[14] Hof K., Lippeert K.M., Schreiner P.R. (2012). Science of Synthesis: Asymmetric Organocatalysis 2.

[15] Knowles R.R., Jacobsen E.N. (2010). Attractive non-covalent interactions in asymmetric catalysis: Links between enzymes and small molecule catalysts. Proc. Natl. Acad. Sci. USA.

[16] Etzenbach-Effers K., Berkessel A., List B. (2009). Non-covalent organocatalysis based on hydrogen bonding: Elucidation of reaction paths by computational methods. Asymmetric Organocatalysis.

[17] De Figueiredo R.M., Mazziotta A., de Sant’Ana D.P., Palumbo C., Gasperi T. (2012). Active methylene compounds in asymmetric organocatalytic synthesis of natural products and pharmaceutical scaffolds. Curr. Org. Chem..

[18] Song C.E. (2009). Cinchona Alkaloids in Synthesis and Catalysis, Ligands, Immobilization and Organocatalysis.

[19] Bai M., Cui B.-D., Zuo J., Zhao J.-Q., You Y., Chen Y.-Z., Xu X.-Y., Zhang X.-M., Yuan W.-C. (2015). Quinine-catalyzed asymmetric domino Mannich-cyclization reactions of 3-isothiocyanato oxindoles with imines for the synthesis of spirocyclic oxindoles. Tetrahedron.

[20] Huang J.R., Sohail M., Taniguchi T., Monde K., Tanaka F. (2017). Formal [4 + 1] cycloaddition and enantioselective Michael-Henry cascade reactions to synthesize spiro[4,5]decanes and spirooxindole polycycles. Angew. Chem. Int. Ed. Eng..

[21] Li T.-Z., Xie J., Jiang Y., Sha F., Wu X.-Y. (2015). Enantioselective vinylogous Michael/cyclization cascade reaction of acyclic β,γ-unsaturated amides with isatylidene malononitriles: Asymmetric construction of spirocyclic oxindoles. Adv. Synth. Catal..

[22] Xie J., Xing X.Y., Sha F., Wu Z.Y., Wu X.Y. (2016). Enantioselective synthesis of spiro[indoline-3,4′-pyrano[2,3-*c*]pyrazole] derivatives via an organocatalytic asymmetric michael/cyclization cascade reaction. Org. Biomol. Chem..

[23] Zhao K., Zhi Y., Li X., Puttreddy R., Rissanen K., Enders D. (2016). Asymmetric synthesis of 3,3′-pyrrolidinyl-dispirooxindoles via a one-pot organocatalytic Mannich/deprotection/aza-Michael sequence. Chem. Commun..

[24] Connon S.J. (2006). Organocatalysis mediated by (thio)urea derivatives. Chem. Eur. J..

[25] Takemoto Y. (2010). Development of chiral thiourea catalysts and its application to asymmetric catalytic reactions. Chem. Pharm. Bull..

[26] Fang X., Wang C.J. (2015). Recent advances in asymmetric organocatalysis mediated by bifunctional amine-thioureas bearing multiple hydrogen-bonding donors. Chem. Commun..

[27] Sun Y.-L., Wei Y., Shi M. (2017). Applications of chiral thiourea-amine/phosphine organocatalysts in catalytic asymmetric reactions. ChemCatChem.

[28] Reddy Gajulapalli V.P., Vinayagam P., Kesavan V. (2015). Enantioselective assembly of functionalized carbocyclic spirooxindoles using anl-proline derived thiourea organocatalyst. RSC Adv..

[29] Auria-Luna F., Marques-Lopez E., Mohammadi S., Heiran R., Herrera R.P. (2015). New organocatalytic asymmetric synthesis of highly substituted chiral 2-oxospiro-[indole-3,4′-(1′,4′-dihydropyridine)] derivatives. Molecules.

[30] Okino T., Hoashi Y., Takemoto Y. (2003). Enantioselective michael reaction of malonates to nitroolefins catalyzed by bifunctional organocatalysts. J. Am. Chem. Soc..

[31] Xie J., Xing W.-L., Sha F., Wu X.-Y. (2016). Enantioselective cascade reaction of α-cyano ketones and isatylidene malononitriles: Asymmetric construction of spiro[4h-pyran-oxindoles]. Eur. J. Org. Chem..

[32] Cui L.Y., Wang Y.M., Zhou Z.H. (2016). Enantioselective construction of novel chiral spirooxindoles incorporating a thiazole nucleus. RSC Adv..

[33] Pratap Reddy Gajulapalli V., Lokesh K., Vishwanath M., Kesavan V. (2016). Organocatalytic construction of spirooxindole naphthoquinones through Michael/hemiketalization using L-proline derived bifunctional thiourea. RSC Adv..

[34] Yin S.-J., Zhang S.-Y., Zhang J.-Q., Sun B.-B., Fan W.-T., Wu B., Wang X.-W. (2016). Organocatalytic tandem enantioselective Michael-cyclization of isatin-derived β,γ-unsaturated α-ketoesters with 3-hydroxy-4h-chromen-4-one or 2-hydroxy-1,4-naphthoquinone derivatives. RSC Adv..

[35] Zhao K., Zhi Y., Shu T., Valkonen A., Rissanen K., Enders D. (2016). Organocatalytic domino oxa-Michael/1,6-addition reactions: Asymmetric synthesis of chromans bearing oxindole scaffolds. Angew. Chem. Int. Ed..

[36] Chowdhury R., Kumar M., Ghosh S.K. (2016). Organocatalyzed enantioselective Michael addition/cyclization cascade reaction of 3-isothiocyanato oxindoles with arylidene malonates. Org. Biomol. Chem..

[37] Wu C., Jing L., Qin D., Yin M., He Q. (2016). Organocatalytic asymmetric synthesis of *trans*-configured trispirooxindoles through a cascade Michael-cyclization reaction. Tetrahedron Lett..

[38] Zhao J.Q., Zhou M.Q., Wu Z.J., Wang Z.H., Yue D.F., Xu X.Y., Zhang X.M., Yuan W.C. (2015). Asymmetric Michael/cyclization cascade reaction of 3-isothiocyanato oxindoles and 3-nitroindoles with amino-thiocarbamate catalysts: Enantioselective synthesis of polycyclic spirooxindoles. Org. Lett..

[39] Yi W.-B., Zhang Z., Huang X., Tanner A., Cai C., Zhang W. (2013). One-pot fluorination and asymmetric Michael addition promoted by recyclable fluorous organocatalysts. RSC Adv..

[40] Huang X., Pham K., Yi W., Zhang X., Clamens C., Hyatt J.H., Jasinsk J.P., Tayvah U., Zhang W. (2015). Recyclable organocatalyst-promoted one-pot asymmetric synthesis of spirooxindoles bearing multiple stereogenic centers. Adv. Synth. Catal..

[41] Huang X., Liu M., Pham K., Zhang X., Yi W.B., Jasinski J.P., Zhang W. (2016). Organocatalytic one-pot asymmetric synthesis of thiolated spiro-γ-lactam oxindoles bearing three stereocenters. J. Org. Chem..

[42] Zhang W., Curran D.P. (2006). Synthetic applications of fluorous solid-phase extraction (f-spe). Tetrahedron.

[43] Enders D., Zhi Y., Zhao K., von Essen C., Rissanen K. (2017). Thiourea-catalyzed domino Michael–Mannich [3 + 2] cycloadditions: A strategy for the asymmetric synthesis of 3,3′-pyrrolidinyl-dispirooxindoles. Synlett.

[44] Monari M., Montroni E., Nitti A., Lombardo M., Trombini C., Quintavalla A. (2015). Highly stereoselective [4 + 2] and [3 + 2] spiroannulations of 2-(2-oxoindolin-3-ylidene)acetic esters catalyzed by bifunctional thioureas. Chem. Eur. J..

[45] Cerisoli L., Lombardo M., Trombini C., Quintavalla A. (2016). The first enantioselective organocatalytic synthesis of 3-spiro-α-alkylidene-gamma-butyrolactone oxindoles. Chem. Eur. J..

[46] Quintavalla A., Lanza F., Montroni E., Lombardo M., Trombini C. (2013). Organocatalytic conjugate addition of nitroalkanes to 3-ylidene oxindoles: A stereocontrolled diversity oriented route to oxindole derivatives. J. Org. Chem..

[47] Muthusamy S., Prakash M., Ramakrishnan C., Gromiha M.M., Kesavan V. (2016). Organocatalytic enantioselective assembly of spirooxindole-naphthopyrans through tandem Friedel-Crafts type/hemiketalization. ChemCatChem.

[48] Vishwanath M., Vinayagam P., Gajulapalli V.P.R., Kesavan V. (2016). Asymmetric organocatalytic assembly of oxindoles fused with spiro-3,4-dihydropyrans with three contiguous stereocenters consisting of vicinal quaternary centers. Asian J. Org. Chem..

[49] Du D., Jiang Y., Xu Q., Tang X.-Y., Shi M. (2015). Enantioselective [3 + 2] cyclization of 3-isothiocyanato oxindoles with trifluoromethylated 2-butenedioic acid diesters. ChemCatChem.

[50] Cook A.G., Voges A.B., Kammarath A.E. (2001). Aminal-catalyzed isomerization of and addition to dimethyl maleate. Tetrahedron Lett..

[51] Malerich J.P., Hagihara K., Rawal V.H. (2008). Chiral squaramide derivatives are excellent hydrogen bond donor catalyst. J. Am. Chem. Soc..

[52] Zhao B.-L., Du D.-M. (2015). Organocatalytic enantioselective cascade aza-Michael/Michael addition sequence for asymmetric synthesis of chiral spiro[pyrrolidine-3,3′-oxindole]s. Asian J. Org. Chem..

[53] Zhao B.L., Du D.M. (2016). Organocatalytic cascade Michael/Michael reaction for the asymmetric synthesis of spirooxindoles containing five contiguous stereocenters. Chem. Commun..

[54] Zhao B.-L., Du D.-M. (2016). Squaramide-catalyzed enantioselective cascade approach to bispirooxindoles with multiple stereocenters. Adv. Synth. Catal..

[55] Chen L., Wu Z.J., Zhang M.L., Yue D.F., Zhang X.M., Xu X.Y., Yuan W.C. (2015). Organocatalytic asymmetric Michael/cyclization cascade reactions of 3-hydroxyoxindoles/3-aminooxindoles with α,β-unsaturated acyl phosphonates for the construction of spirocyclic oxindole-γ-lactones/lactams. J. Org. Chem..

[56] Ming S., Zhao B.L., Du D.M. (2017). Chiral squaramide-catalysed enantioselective Michael/cyclization cascade reaction of 3-hydroxyoxindoles with α,β-unsaturated *N*-acylated succinimides. Org. Biomol. Chem..

[57] Lin Y., Liu L., Du D.-M. (2017). Squaramide-catalyzed asymmetric Michael/cyclization cascade reaction of 3-isothiocyanato oxindoles with chalcones for synthesis of pyrrolidinyl spirooxindoles. Org. Chem. Front..

[58] Liu L., Zhao B.-L., Du D.-M. (2016). Organocatalytic asymmetric Michael/cyclization cascade reaction of 3-isothiocyanato oxindoles with maleimides for the efficient construction of pyrrolidonyl spirooxindoles. Eur. J. Org. Chem..

[59] Lou Q., Ding Y., Xu D., Liu G., Zhao J. (2017). Organocatalytic enantioselective synthesis of dihydropyrano- indole derivatives bearing trifluoromethylated all-carbon-substituted stereocenters. Adv. Synth. Catal..

[60] Curti C., Rassu G., Zambrano V., Pinna L., Pelosi G., Sartori A., Battistini L., Zanardi F., Casiraghi G. (2012). Bifunctional cinchona alkaloid/thiourea catalyzes direct and enantioselective vinylogous Michael addition of 3-alkylidene oxindoles to nitroolefins. Angew. Chem. Int. Ed. Engl..

[61] Rassu G., Zambrano V., Pinna L., Curti C., Battistini L., Sartori A., Pelosi G., Zanardi F., Casiraghi G. (2013). Direct regio-, diastereo-, and enantioselective vinylogous Michael addition of prochiral 3-alkylideneoxindoles to nitroolefins. Adv. Synth. Catal..

[62] Rassu G., Zambrano V., Tanca R., Sartori A., Battistini L., Zanardi F., Curti C., Casiraghi G. (2012). 3-alkenyl-2-silyloxyindoles: An enabling, yet understated progeny of vinylogous carbon nucleophiles. Eur. J. Org. Chem..

[63] Han J.L., Chang C.H. (2016). An asymmetric assembly of spirooxindole dihydropyranones through a direct enantioselective organocatalytic vinylogous aldol-cyclization cascade reaction of 3-alkylidene oxindoles with isatins. Chem. Commun..

[64] Zhao Y.L., Lou Q.X., Wang L.S., Hu W.H., Zhao J.L. (2017). Organocatalytic Friedel-Crafts alkylation/lactonization reaction of naphthols with 3-trifluoroethylidene oxindoles: The asymmetric synthesis of dihydrocoumarins. Angew. Chem. Int. Ed. Eng..

[65] Huang L.-J., Weng J., Wang S., Lu G. (2015). Organocatalytic diels-alder reaction of 2-vinylindoles with methyleneindolinones: An efficient approach to functionalized carbazolespirooxindoles. Adv. Synth. Catal..

[66] Shi F., Zhang H.H., Sun X.X., Liang J., Fan T., Tu S.J. (2015). Organocatalytic asymmetric cascade reactions of 7-vinylindoles: Ddiastereo- and enantioselective synthesis of C7-functionalized indoles. Chem. Eur. J..

